# XNOR-Nets with SETs: Proposal for a binarised convolution processing elements with Single-Electron Transistors

**DOI:** 10.1038/s41598-022-13180-7

**Published:** 2022-06-15

**Authors:** Varun Bheemireddy

**Affiliations:** Device Modeling Group, James Watt School of Engineering, The University of Glasgow, Glasgow, G12 8QQ UK

**Keywords:** Electrical and electronic engineering, Nanoscience and technology, Nanoscale devices

## Abstract

Deep neural network (DNN) and Convolution neural network (CNN) algorithms have significantly increased the accuracies in cutting-edge large-scale image recognition and natural-language processing tasks. Generally, such neural nets are implemented on power-hungry GPUs, beyond the reach of low-power edge-devices. The binary neural nets have been proposed recently, where both the input activations and weights are constrained to $+$ 1 and − 1 to address this challenge. Here in the present proof-of-concept study, we propose a simple class of mixed-signal circuits composed of single-electron devices and exploit the nonlinear Coulomb staircase phenomena to alleviate the challenges of binarised deep learning hardware accelerators. In particular, through SPICE modeling, we demonstrate the realisation of space-time-energy efficient XNOR-Accumulation (XAC) operation, reconfigurabilty of XAC circuit to perform 1D convolution and a busbar design to augment a contemporary accelerator. These nanoscale circuits could be readily fabricated and may potentially be deployed in low-power deep-learning systems.

## Introduction

Deep neural network (DNN) and Convolution neural network (CNN) algorithms have dramatically increased the target accuracies in cutting-edge large-scale image recognition tasks^1–4^. But, such networks are beyond the range of mW-level low-power systems and are trained exclusively on power-hungry and massively parallel GPUs. To address this energy problem and bring edge-computing closer to practicality, algorithms have been proposed to constrain both the input activations and weights to $+$ 1 and − 1^5,6^. These binary neural nets greatly reduces the memory size by 32x and replace the costly multiply-accumulate (MAC) operations by a simpler XNOR-Accumulate (XAC) computation, still maintaining a respectable accuracy relative to full-precision neural nets. At the architectural level, further reduction of the energy consumption due to data movement between computing unit and memory unit could be achieved by in-memory analog computation^7^. In these architectures, multiplication is performed by encoding non-volatile memory conductance as the weight parameter, the voltage pulse as the input and the output current as the product. The addition is performed by the summation of currents governed by the Kirchhoff’s law of current. But these weight-stationary analog computing devices faces a roadblock in terms of writing and reading speeds, significant energy costs to write the weight parameter, endurance, variability issues and non-linear conductance updates^8^. The mixed-signal approach has been proposed^9^ that utilises energy-efficient switched-capacitor neuron circuit design to sum the XNOR products in an analog fashion. This proof-of-concept ASIC achieved 3.8 $\upmu$J/classification at 86% accuracy on the CIFAR-10 image classification data set with a 28-nm CMOS running at 0.6 V/0.8 V voltage supplies.

Single-electron transistor (SET) device is a matured technology that predates the current dominant landscape of non-volatile memory devices employed in in-memory computation and shows great promise in the conventional digital computing^10^ and quantum computing^11,12^. SET shows remarkable nanoscale physical phenomena of Coulomb oscillations, Kondo physics and Coulomb staircase^13^. Single-electron phenomena were also observed in a range of low-dimensional systems, of which novel 2D materials became the new entrants recently^14,15^. The hybrid circuits of SET-FET are demonstrated as a post-CMOS solution and also to complement the inadequacies of SETs^16,17^.

In this study, we propose a simple mixed-signal circuit design utilising the Coulomb stair case phenomenon of SET to alleviate few of the above mentioned problems in the hardware acceleration of binarised deep neural nets. In particular, we demonstrate through SPICE modeling, the XNOR-Accumulation (XAC) operation obtained from a simple design of SET-based circuit that show huge gains in space-time-energy resources. Then we proceed on to reconfigure the XAC circuit to carry out the 1D convolution by taking a simple test case of $4\times 4$ kernel convolution with input activations. Though the reconfigurability saves the chip area, it has its own fair share of disadvantages as discussed in the relevant section. Thus, we provide a solution with the simple busbar circuit of two-terminal SETs and also show the integrability of busbar into a contemporary binary neural net accelerator for further acceleration.

## Results

**Figure 1:**
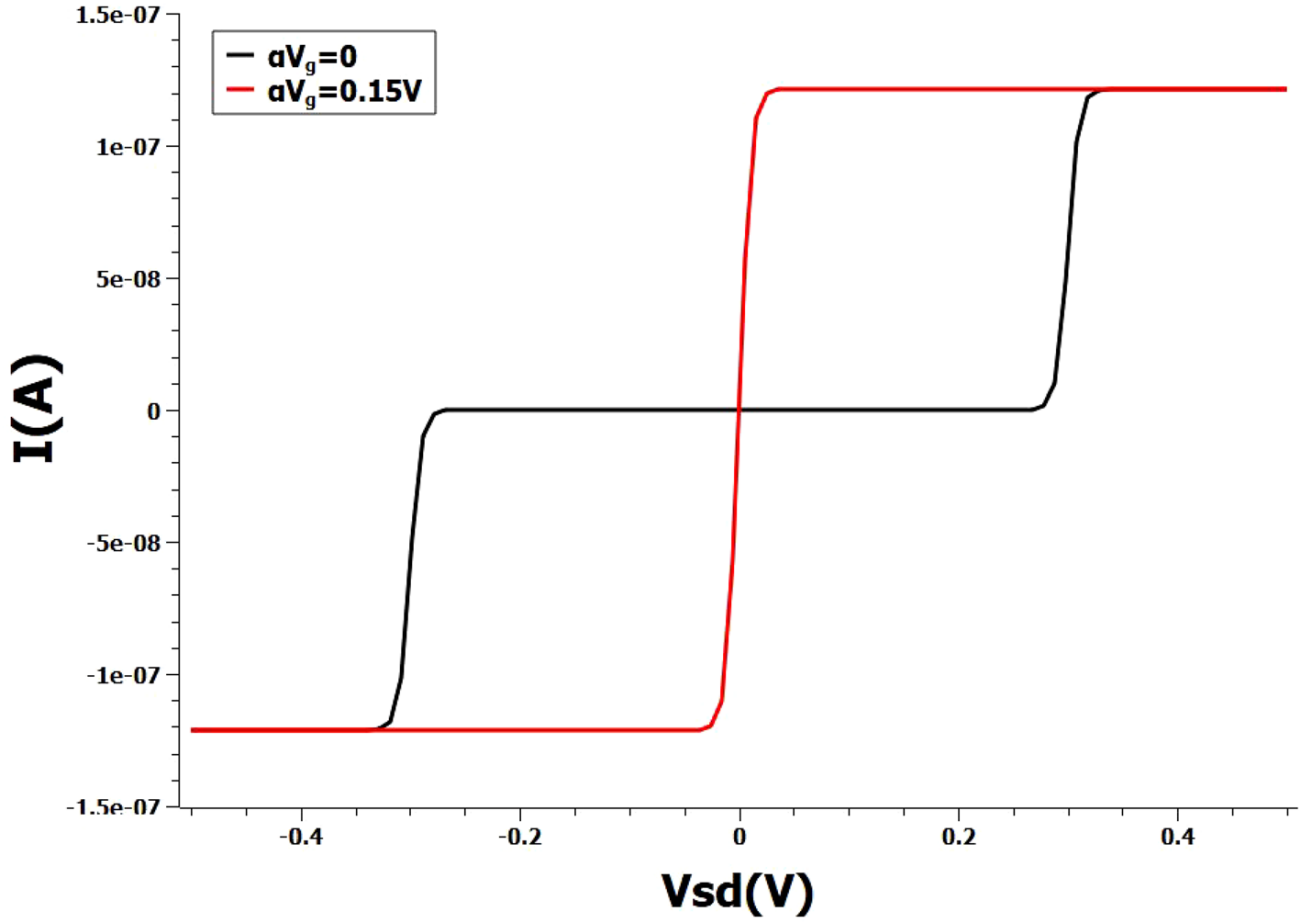
Non-linear Coulomb staircase current-voltage curve of a single-level model. The analytical curves are obtained for arbitrary gate coupling factor $\alpha$ and the gate voltages include the coupling factor. Black curve represent zero gate voltage, Red curve represent scaled gate voltage of 0.15 V.

Figure 1 shows the typical Coulomb staircase behaviour of current-voltage characteristics for an one level single-electron system^13^. The results are obtained for a simple one-level device at two gate voltages, Vg = 0 V and Vg = 0.15 V. The current-voltage curves are obtained by analytically solving the Master equation for a single energy state. The initial point of the problem is to implement AND operation using the non-linear Coulomb staircase of a single SET. We use this one-level toy model to briefly discuss about the implementation of AND. Here, the two inputs are encoded as voltages of source and gate respectively and the output is encoded as the current. AND gate could be realised by encoding input logical **0** and **1** as voltages 0 V and 0.15 V respectively and output logical **0** and **1** as 0 A and 1.2e−7 A respectively. For example, inputs **0** and **0** gives output logic **0** and similarly, inputs **1** and **1** give output logic **1**.

**Figure 2:**
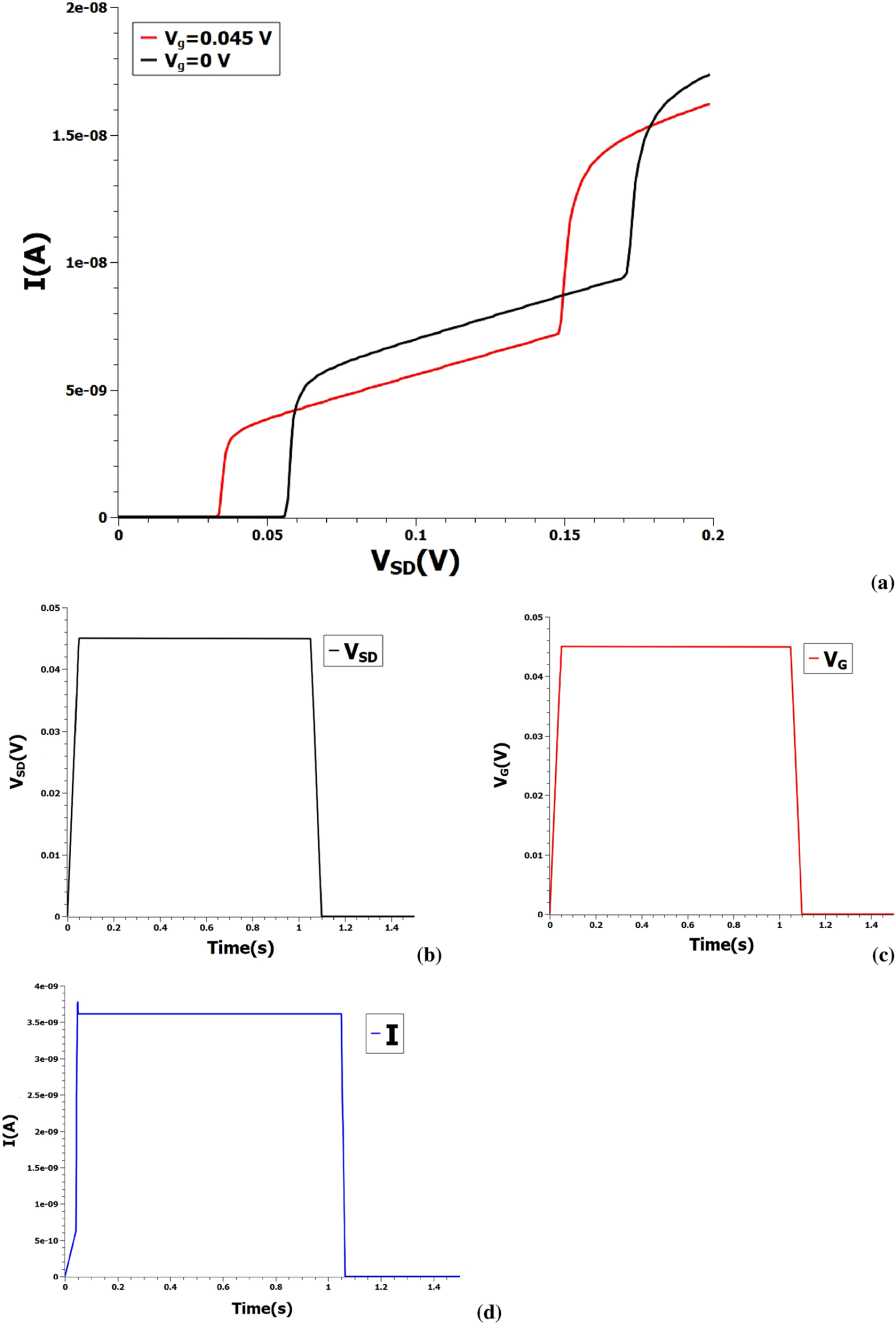
(**a**) Current–voltage characteristics of single-electron transistor at two gate voltages of 0 V and 0.045 V as obtained from SPICE modeling. (**b**, **c**) Two input voltage pulses measured at 0.045 V fed into the AND gate. (**d**) The resultant output current pulse obtained at 3.6 nA.

With the basic philosophy of the problem laid down using a simple one-level model, we proceed on to a detailed study of the problem with a more rigorous Master equation based SPICE model^18^. Figure 2a depicts the typical Coulomb-staircase behaviour of a single-electron transistor obtained from SPICE-level simulation at gate voltages of 0 V and 0.045 V. The operation of AND as described above is illustrated in Fig. 2 taking an example input instance of **1** and **1**.The output current measured at 3.6 nA is the logical output **1** for the given inputs. The inputs **0** and **1** of the Boolean gate could be set at the Coulomb levels of choice depending on the drive current and energy trade-off required in the application-specific circuit design. The current levels also offer high robustness to input voltage noises due to the Coulomb blockade of electrons. Moreover, the sensing margin of outputs namely I(**1**)/I(**0**) has an ideal value of infinity, in principle.

**Figure 3:**
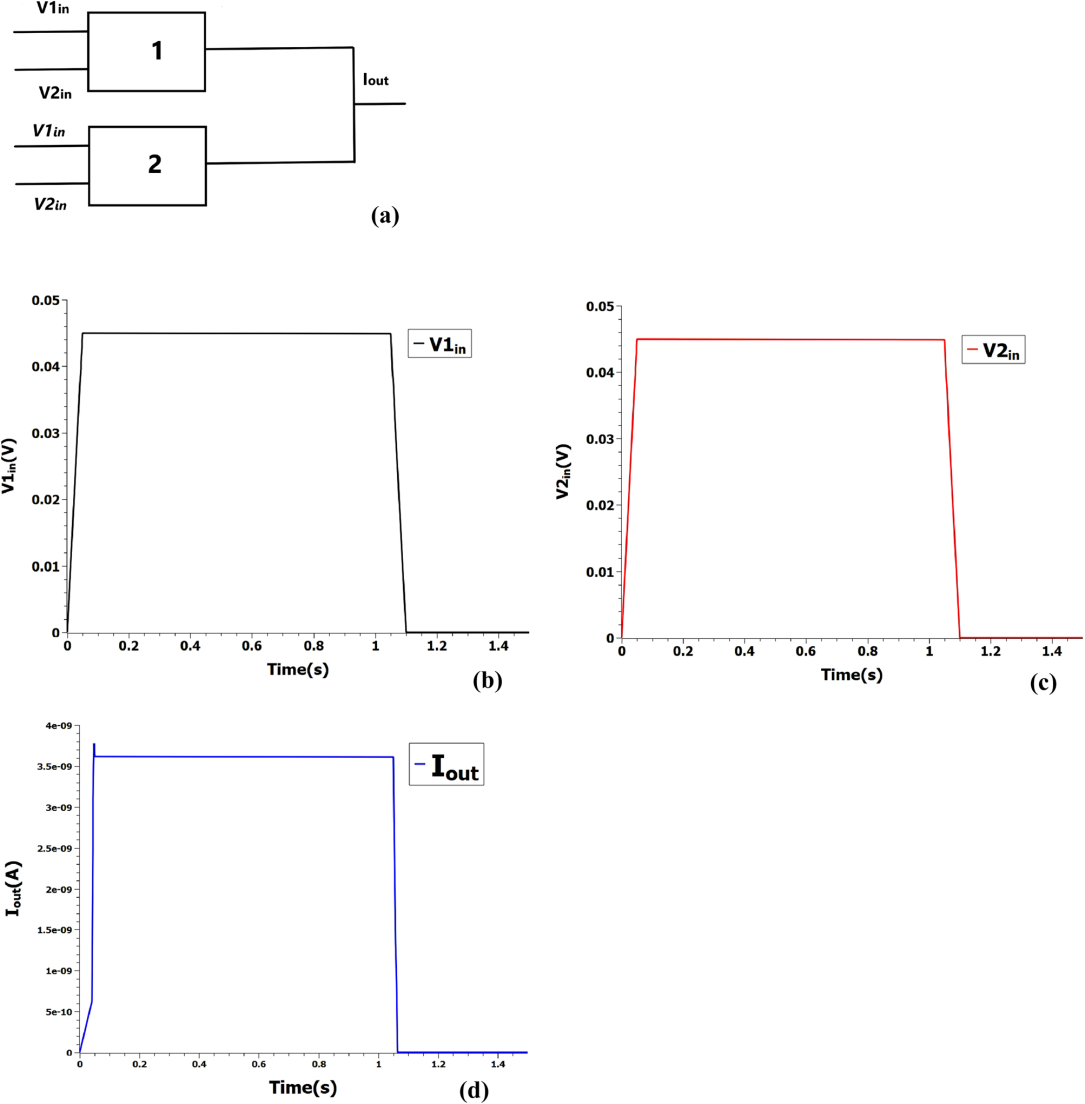
(**a**) XNOR operation from two AND and one OR operation. 1 and 2 represent SET. Italicised voltages represent corresponding inverted logic values. (**b**, **c**) Two input voltage pulses measured at 0.045 V fed into the XNOR gate. (**d**) The resultant output current pulse is measured at 3.6 nA.

In Binary Neural Nets, the input activations and weights are binarised to either $+$ 1 or − 1 and therefore, n-bit floating point Multiply-Accumulate operations are transformed to XNOR-Accumulate^5^. XAC remains the core operation of the binary deep neural networks consuming the bulk of space, time and energy resources. XNOR operation is derived from the AND operation by adding a second SET and inverting the input voltage signals as implemented by Eq. 1 and shown in Fig. 3. XNOR and XOR operations are also obtained in the previous studies^19,20^ using a similar input and output encoding scheme but with a more complicated multi-gate device design and fabrication process.

1 $$\begin{aligned} \mathbf{p} \quad XNOR \quad \mathbf{q} = ( \mathbf{p} \quad AND \quad \mathbf{q} ) \quad OR \quad ( \overline{\mathbf{p }} \quad AND \quad \overline{\mathbf{q }} ) \end{aligned}$$

### XNOR and accumulate (XAC)

**Figure 4:**
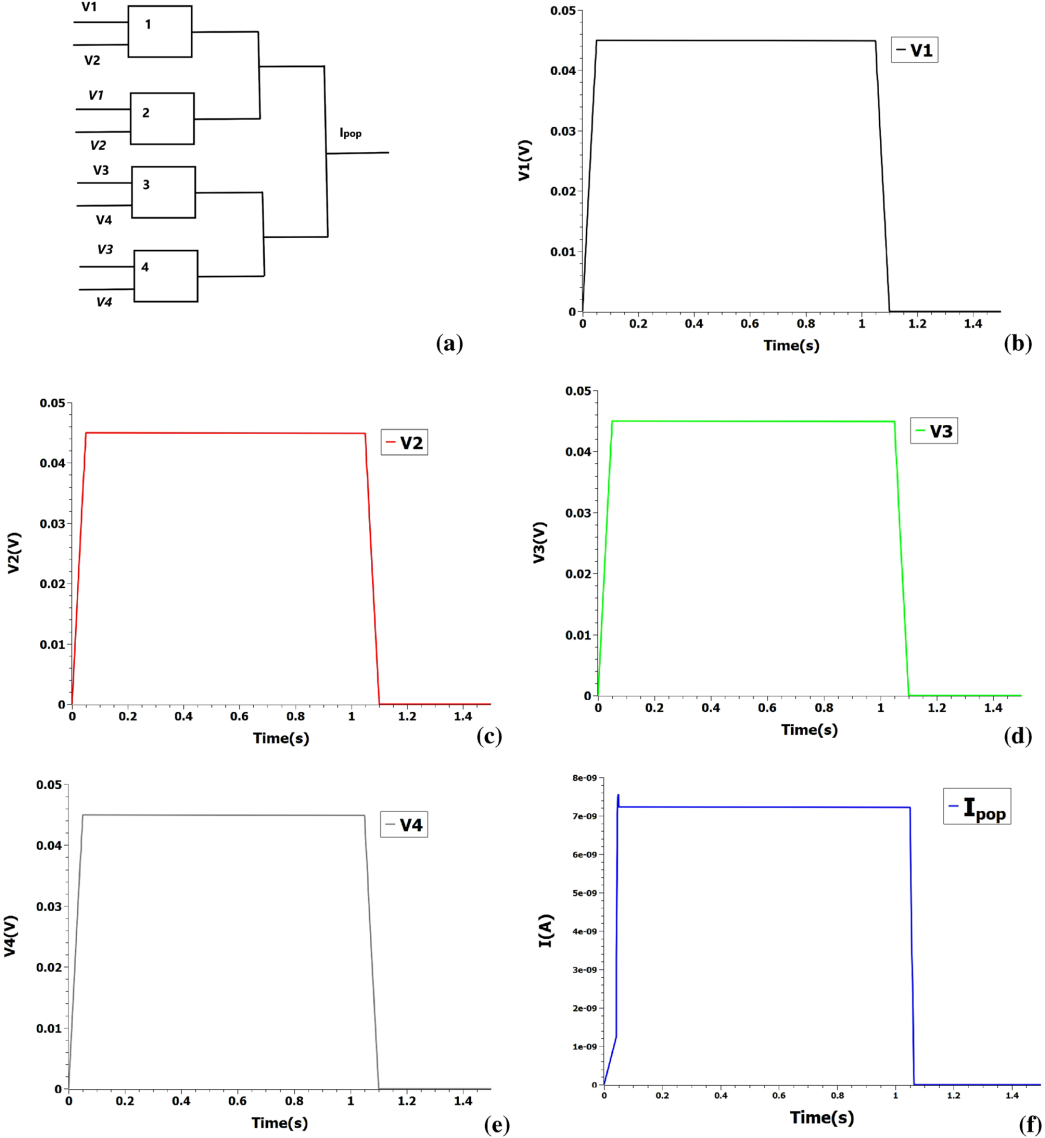
(**a**) XAC circuit to perform XNOR and POPCOUNT operations. 1, 2, 3, 4 represent SETs. Italicised voltages represent corresponding inverted logic values. Ipop represent the POPCOUNT value. (**b**–**e**) Input voltage pulses at 0.045 V encoding logic **1** are loaded into XAC. (**f**) POPCOUNT value is measured at output current 7.2 nA encoding integer 2.

The accumulation is done by POPCOUNT instruction in digital systems and consumes vast resources of space and time for big-length inputs. The XAC circuit that builds on the previous XNOR circuit is shown in Fig. 4a that POPCOUNT the 2-bit length input. The circuit uses the Kirchhoff’s law of current to POPCOUNT the input. Long Cheng et al.^21^ experimentally demonstrated a 4-bit POPCOUNT accelerator using a memristive array that similarly operates on the addition of currents. For illustration of the POPCOUNT operation, consider two binarised inputs A = [1 1] and B = [1 1]. The first bits of A and B are fed into V1 and V2, while second bits are fed into V3 and V4. SETs numbered 1,2,3 and 4 perform XNOR operation on the vectors and the POPCOUNT is read by measuring the output current Ipop. In the present example, the input voltages are fixed at 0.045 V and the output current is measured at 7.2 nA which is encoded as integer 2 (Fig. 4).

### Reconfigurable XAC circuit

**Figure 5:**
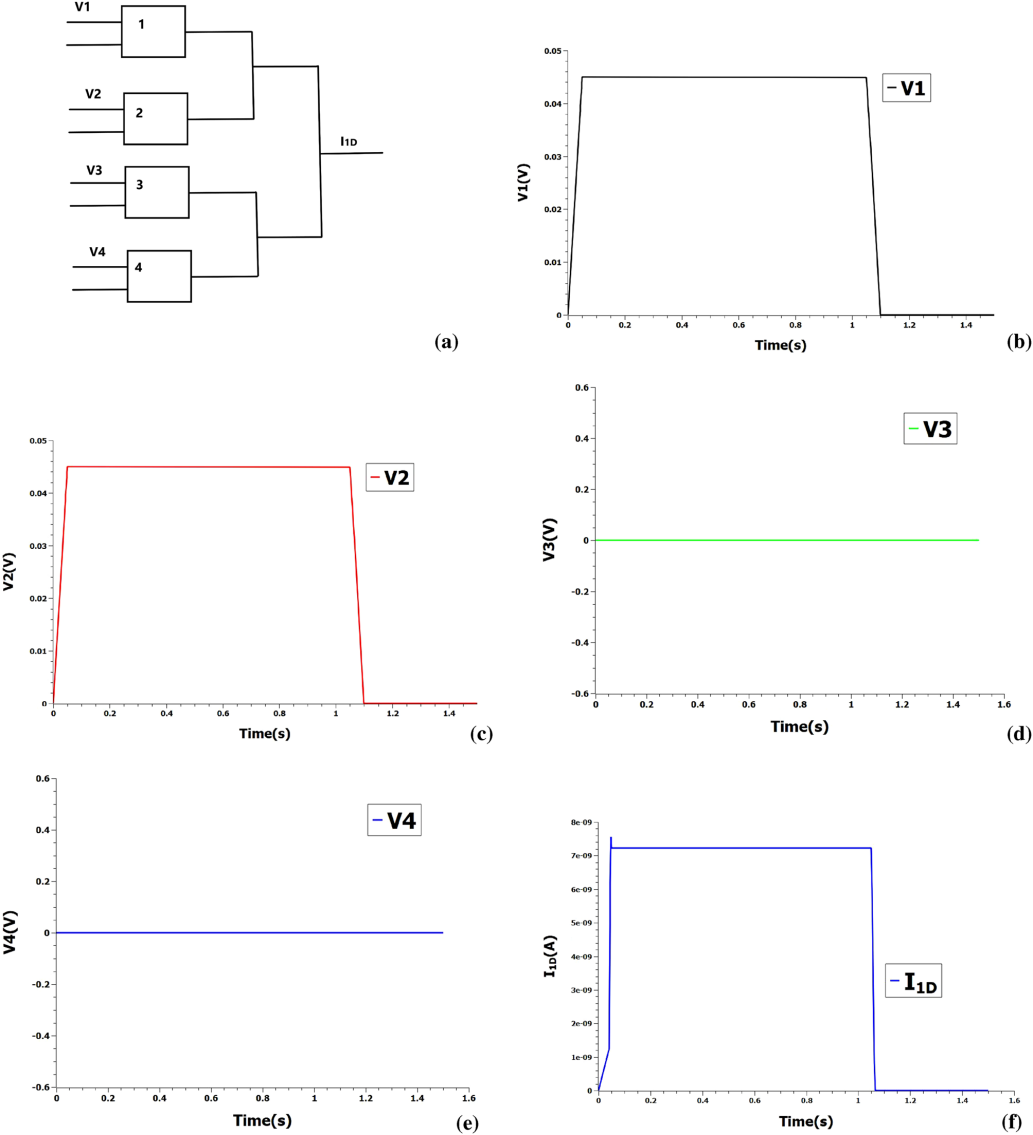
(**a**) Reconfigured XAC to perform 1D row convolution. 1, 2, 3, 4 represent SETs. The gate voltage is fixed at 0.045 V. $I_{1D}$ represent the 1D convolution value. (**b**–**e**) Input voltage pulses at 0.045 V and 0 V encoding POPCOUNT integer values of 1 and 0 respectively are loaded into reconfigured XAC. (**f**) 1D convolution value is measured at output current 7.2 nA encoding integer 2.

The advantage of the proposed XAC circuit is that it could be reconfigured to carry out the 1D and 2D convolutions^7^ for a parallel binarised CNNs. Here, we demonstrate the reconfigurability of XAC circuit by taking a example of performing 1D row convolution of $4\times 4$ kernel with input activations. Once the POPCOUNT operation as implemented by the XAC unit is completed, the POPCOUNT outputs of different XAC units are routed back to a selected XAC unit to obtain 1D convolution output as shown in Fig. 5a. The gate terminals could be grounded or fixed at selected voltage and therefore, the devices function essentially as two-terminal single-electron junctions for the 1D convolution operation. POPCOUNT is encoded at voltages that maps to equivalent Coulomb stair-case level. Each quantised current level maps to an unique integer obtained from the POPCOUNT instruction. The resultant output current obtained by the summation of individual devices represent the 1D convolution value. Both positive and negative sums could be computed by encoding the sign in the direction of current. For example, assume the POPCOUNTs obtained from XAC of one row of $4\times 4$ kernel with input activations to be V1 = 1, V2 = 1, V3 = 0 and V4 = 0. These integers are fed into the reconfigured XAC unit, with the voltages fixed at 0.045 V and 0 V to represent integer 1 and 0 respectively. The output current, $I_{1D}$ measured at 7.2 nA encode the 1D convolution value 2 as shown in Fig. 5. It should be noted that current should be converted to appropriate voltage level with an additional converter circuit before feeding inputs into the reconfigured XAC circuit^22^.

### Busbar circuit

The re-configurable XAC circuit provides a significant savings in transistor consumption and the chip area. But it is achieved at the expense of complex three-terminal transistor fabrication, delayed computation and importantly, integrating the SET-XAC circuit with other contemporary deep neural hardware could be challenging. Therefore, a simpler busbar circuit consisting of two-terminal single-electron junctions is proposed to address this problem. The crossbar of silicon quantum dots has already been demonstrated^23^ for a more complicated architecture in quantum computing. In particular, we demonstrate the 1D convolution operation of the busbar circuit by integrating into and augmenting a contemporary hardware accelerator, XNORBIN^24^. XNORBIN, a completely digital and tapeout ASIC achieved the second-best result of 100 TOP/s/W designed on a Global Foundries 65nm node.

**Figure 6:**
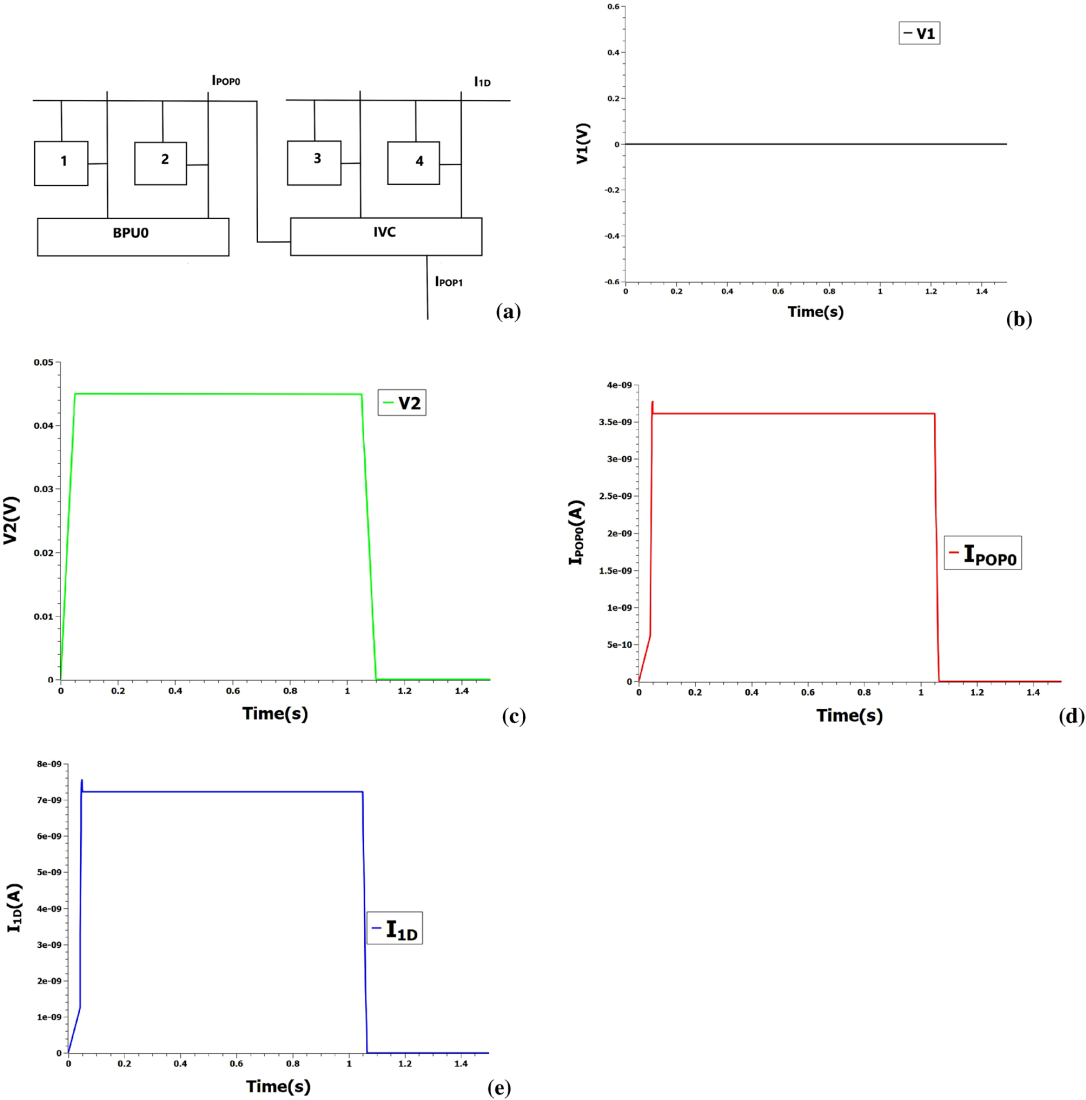
(**a**) The busbar circuit composed of two-terminal single-electron junctions numbered 1, 2, 3, 4. BPU0 module provides the output of XNOR operation. IVC represent current-to-voltage converter circuit. $I_{POP0}$ and $I_{POP1}$ denote the POPCOUNT values from busbars. $I_{1D}$ denotes the final 1D convolution value. (**b**, **c**) Input voltage pulses at 0 V and 0.045 V encoding XNOR output **0** and **1** respectively of BPU0 are loaded into the busbar. (**d**, **e**) Ouput POPCOUNT from the first busbar and 1D convolution value from the second busbar are measured at output currents 3.6 nA and 7.2 nA encoding the integers 1 and 2 respectively.

Here, we use a simple two-element busbar to demonstrate our proof-of-concept as shown in Fig. 6a and the present design could be scaled to arbitrary length. POPCOUNT and full-adder units of Basic processing unit (BPU) of XNORBIN are replaced with two busbars and the outputs of BPU XNOR ( voltage scaled appropriately ) are fed into the inputs of busbar. Since the busbar element length is 2, the input and size of the kernel is restricted to 2-bit vector and $2\times 2$ respectively for our proof-of-concept illustration. Consider the outputs of XNOR to be 0 and 1 in both the BPUs (BPU0 and BPU1). As shown in Fig. 6, the output current $I_{POP0}$ ( POPCOUNT from BPU0 = 1 ) generated from two-terminal single-electron junctions 1 and 2 of first busbar is fed into current-to-voltage converter (concomitantly POP1 from BPU1 = 1 is also fed ) to generate the appropriate input voltages for the second busbar. The POPCOUNT inputs are loaded onto the two-terminal single-electron junctions 3 and 4 of second busbar to get 1D convolution output of value 2. In principle, the complex digital adder circuits of XNORBIN could be replaced by a simple analog busbars to further accelerate the deep neural convolution nets.

For a methodical performance analysis of our convolution processing elements, a rigorous system-level performance is required accounting for interconnects, current-sensing amplifiers, current-controlled voltage sources and is beyond the scope of present work. Nevertheless, an attempt is made to reasonably compare the SET based computation and the digital baseline taking fundamental XNOR and Full-adder(FA) operations on a 2-bit 2 inputs as the relevant parameter and tabulated in Table 1. Here, the comparison is benchmarked against a fully digital circuit^25^ taking only the design that performed best in Power-Delay-Product(PDP) metrics. All the digital circuits have been designed on a 65 nm TSMC CMOS process technology node. The proposed full-swing XNOR gate consists of 7 transistors with the best PDP metric of 52.9 aJ. The FA, with the best PDP metric of 241.1 aJ, is a 22-transistor configuration that implements a well-known four-transistor 2-1-MUX structure. SET-based inverters^26^ are considered to drive inverted inputs into the SET-XAC unit and the worst-case performance metrics of the XAC is reported. The parameters of the remaining SETs are also assumed to have similar values as SET-inverters. The SET based unit shows 66% lesser transistor utilisation, nearly-equivalent speed of XAC operation and an almost 4-order magnitude reduction in power dissipation at 1 GHz clock frequency.

**Table 1:** Performance comparison of the proposed SET-based unit with a XNOR plus FA digital system.

Performance parameter	Digital circuit	SET-based unit
Transistor usage	36	12
Delay (ns)	0.08	$\approx 0.1$
Power consumption ($\mu$ W) at 1 GHz	9	0.001

## Discussion

In summary, we demonstrate a SPICE-modeled simple mixed-signal single-electron transistor based circuits which capitalises on Coulomb staircase current–voltage characteristics, to possibly accelerate the binary deep neural nets. The present circuit design and the results could be readily fabricated and realised, on the condition that the maturity of SET fabrication be able to tackle reliability issues and the current proposal may find potential application either independently or complementing other ASICs in future low-power deep-learning devices.

## Methods

To obtain analytical solution of one-level model, the following physical parameters are used: Right and Left tunneling rates = 1 meV, Single state energy = 50 meV, Charging energy = 100 meV, Temperature = 30 K.

SPICE model parameters are as follows: Circuit simulations were carried out using freeware LTSPICE (https://www.linear.com/solutions/1066). Capacitance of all junctions is fixed at 0.7 aF. Resistance of source junction = 10 M$\Omega$, Resistance of drain junction = 0.1 M$\Omega$, Temperature = 2 K.

## Data Availability

The datasets generated during and/or analysed during the current study are available from the corresponding author on reasonable request.
